# Trace elements in the *Fontinalis antipyretica* from rivers receiving sewage of lignite and glass sand mining industry

**DOI:** 10.1007/s11356-015-4162-y

**Published:** 2015-02-03

**Authors:** Grzegorz Kosior, Aleksandra Samecka-Cymerman, Krzysztof Kolon, Anna Brudzińska-Kosior, Waldemar Bena, Alexander J. Kempers

**Affiliations:** 1 Department of Ecology, Biogeochemistry and Environmental Protection, Wrocław University, ul. Kanonia 6/8, 50-328 Wrocław, Poland; 2 Society of Nature and Landscape Protection, Olszewskiego 7, 59-900 Zgorzelec, Poland; 3 Department of Environmental Science, Institute for Water and Wetland Research, Radboud University Nijmegen, Heyendaalseweg 135, 6525 AJ Nijmegen, The Netherlands

**Keywords:** Aquatic moss, Metal, Bioindication, Mining industry

## Abstract

Intensive lignite and glass sand mining and industrial processing release waste which may contain elements hazardous to the aquatic ecosystem and constitute a potential risk to human health. Therefore, their levels must be carefully controlled. As a result, we examined the effects of sewage on the aquatic *Fontinalis antipyretica* moss in the Nysa Łużycka (lignite industry) and the Kwisa Rivers (glass sand industry). The Nysa Łużycka and the Kwisa Rivers appeared to be heavily polluted with As, Cd, Co, Cr, Cu, Fe, Mn, Ni, Pb, V and Zn, which were reflected in the extremely high concentration of these elements in *F. antipyretica* along the studied watercourses. In the Nysa Łużycka, trace element composition in the moss species is affected by lignite industry with accumulation in its tissues of the highest concentrations of Cd, Co, Cr, Cu, Mn, Ni, Pb and Zn, while samples from the Kwisa sites influenced by glass sand industry revealed the highest concentrations of As, V and Fe. The principal component and classification analysis classifies the concentration of elements in the aquatic *F. antipyretica* moss, thus enabling the differentiation of sources of water pollution in areas affected by mining industry.

## Introduction

The metals often present in industrial wastewaters are hazardous to the aquatic ecosystem and may affect the quality of life. Therefore, their elimination from the aquatic environment plays an important role in water pollution control. As observed by Markert et al. (2003) and Vázquez et al. (2004), pollution monitoring using bioaccumulators is one of the methods for the evaluation of xenobiotic levels. Plants accumulate trace elements and provide an indication of their soluble fraction in the surrounding environment which is likely to affect major compartments of the aquatic ecosystem (Rasmussen and Andersen 1999; Kłos et al. 2010; Krems et al. 2013). Owing to these “accumulating bioindicators”, the level of pollutants in the environment can be studied (Remon et al. 2013). Aquatic mosses are commonly considered invaluable, efficient accumulators of pollutants and ideal indicators of metal contamination (de Traubenberg and Ah-Peng 2004; Pekka et al. 2008; Vuori and Helisten 2010; Vázquez et al. 2013; Cesa et al. 2014). Due to their morphological and physiological characteristics, their ability to accumulate xenobiotics and their widespread occurrence, aquatic mosses are very useful in bioindication and biogeochemical prospecting (Bleuela et al. 2005). Aquatic mosses have a greater accumulation capacity for metals than sediment or vascular macrophytes and allow the characterisation of contaminant bioavailability (Fernández et al. 2006; Dazy et al. 2009). These plants gauge the quality of the environment providing an integrated assessment of a typically discontinuous series of contamination events (Siebert et al. 1996). One of the most commonly used taxa for biomonitoring is *Fontinalis antipyretica* Hedw. (Fontinalaceae), a moss widely distributed in the northern hemisphere and shown to accumulate different contaminants at high levels (Bleuela et al. 2005; Vuori and Helisten 2010; Díaz et al. 2013). This species lacks a well-developed cuticle and vascular tissue, which enables metal uptake directly from water by adsorption and absorption through cell surfaces (Davies 2007; Vuori and Helisten 2010). *F. antipyretica* has a capacity to characterise the quality of sewage-affected water, which may give an indication of the level of ecological risk due to pollution (Vázquez et al. 2013).

The biomass of this aquatic moss can be used as a sorbent for the purification of metal-bearing waste water (Martins et al. 2004; Rau et al. 2007). Many natural rivers have been exposed to metal contamination from anthropogenic sources. In this investigation, two rivers were selected: the Nysa Łużycka polluted with Bogatynia lignite industry sewage and the Kwisa receiving effluents from the Osiecznica glass sands mine. Lignite mining industry is a major source of trace elements and other pollutants influencing ecosystem development (Maiti 2007). The Osiecznica glass sand deposit is enriched with heavy minerals which may be a source of considerable environmental pollution (Łuszczkiewicz 1987). The aim of this study was to investigate the level of contaminants (As, Cd, Co, Cr Cu, Fe, Mn, Ni, Pb, V and Zn) in *F. antipyretica* collected from the two rivers affected by different types of pollution. These results were juxtaposed with similar analyses of water samples collected from the same sites, which allowed us to determine the elemental composition of the water both directly and indirectly, using a bioindicator organism (Vázquez et al. 2004). The tested hypotheses were (1) whether the ubiquitous *F. antipyretica* may be used as a suitable bioindicator of toxic elements in sewage from glass sand processing and mining and from lignite industry and (2) whether principal component and classification analysis classifying the concentration of elements in *F. antipyretica* would enable differentiation of the origin of pollution through relevant patterns.

## Materials and methods

**Fig. 1:**
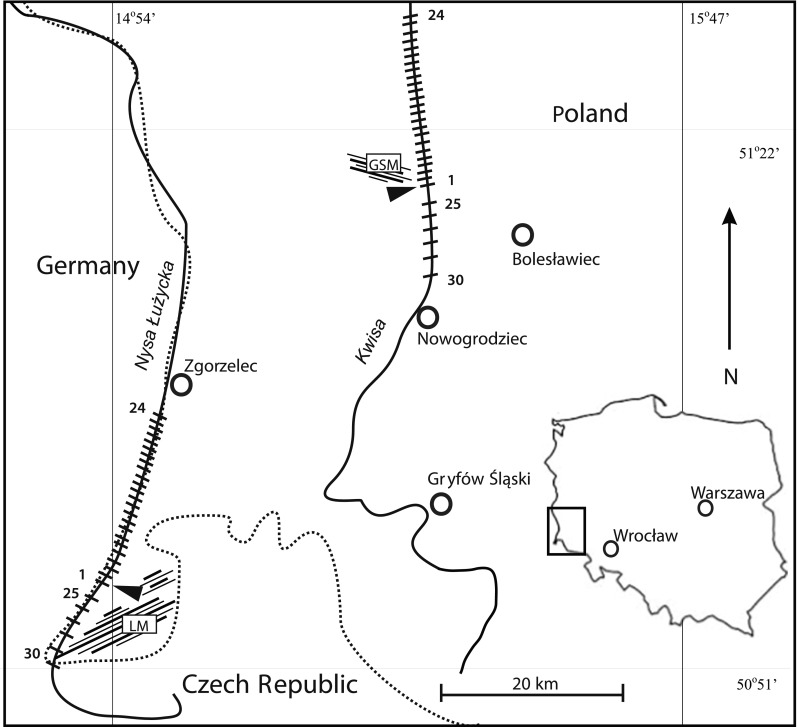
Map showing the sampled section of the Nysa Łużycka and the Kwisa Rivers: ; border: ; sewage output: ; glass sand mine: , lignite mine:

Two rivers in the Lower Silesia (SW Poland) were selected in which *F. antipyretica* occurred naturally (Fig. 1). The Nysa Łużycka (198 km in length) is the second largest left tributary of the Oder. The river flows into Poland from the Czech Republic through the Zittau-Zgorzelec Basin between the Izera Foreland in Poland and the Lusatian Foreland in Germany. Particular characteristic is a narrow gorge of Precambrian granitoids and Neogene basalts. The Nysa Łużycka flows into the Silesian Lowland upstream from Zgorzelec. In the west, the Nysa Łużycka valley is delimited by the Lower Silesian Forest. The main lithology of the Nysa Łużycka consists of fluvial deposits (sands, gravels, muds, peats and organic silts) (Badura and Przybylski 2000; Marks et al. 2006; Badura et al. 2012). The depth of the river is highly variable and ranges from several dozen of centimetres to several metres in the investigated area. River width in the section concerned is approximately 5–10 m. The river is classified in terms of its hydrological regime as a mountain and piedmont river, with flow rate ranging from 0.03 to 16.22 m^3^/s. The catchment area of the Nysa Łużycka is subject to a strong human impact associated with mining activities and intensification of water economy. Thus, the natural water balance is significantly disturbed (Badura and Przybylski 2000). The Kwisa River (140 km in length) is the largest tributary of the Bóbr River. The source of the river lies at an altitude of approximately 900 m above the sea level in the Izera Mountains. The Kwisa flows in the study area through a vast complex of the Lower Silesian Forest. The bottom of the meandering valley comprises typical fluvial deposits (sands, gravels, muds, peats and organic silts). Outcrops of the Upper Cretaceous sandstones, marls and mudstones occur in the southern part of the river (the Kaczawa foreland) (Badura and Przybylski 2000; Marks et al. 2006; Badura et al. 2012). This river has an average width of approximately 15 m, the depth ranges from 0.5 to 1 m in straight sections and 2.5 m in bends with average flow rate ranging from 1.01 to 7.28 m^3^/s (Badura and Przybylski 2000; Machajski 2012). In the case of the two rivers, the climate is temperate with humid air masses from the Atlantic (Najbar et al., 1999). The Nysa Łużycka receives sewage from Bogatynia lignite industry while the Kwisa is polluted by the Osiecznica glass sands mine. In each river, sampling sites were selected at regular intervals starting downstream from the outlet (sites 1–24 spaced 0.83 km from each other) and directly upstream from the sewage outlet (sites 25–30 spaced 2.5 km from each other). Samples of water and *F. antipyretica* were collected from either river on the same day. At each sampling point, three subsamples were taken along a line perpendicular to the river: one subsample in the middle of the river and two subsamples at both sides at a distance from the middle of 1/4 of stream width. Each replicate consisted of a tuft of about 20 g of wet moss. The mosses were washed thoroughly in river water to remove attached particles and epifauna. Basal stems were removed, and apices were cut to a length of 2 cm according to Vázquez et al. (2004). The total number of water and plant samples per river was 30 × 3 = 90.

## Water and plant analysis

Prior to the analysis, the water samples were acidified to pH ≤ 2 with spectrally pure HNO_3_ and filtered through 0.45 μm glass microfiber filters to determine total As and metal concentrations (Ladislas et al. 2012). Three replicates were analysed separately.

The moss samples were dried at 50 °C to constant weight and homogenised to fine powder in an IKA Labortechnik M20 laboratory mill. Dried plant samples (300 mg dry weight, in triplicate) were digested with 3 mL of nitric acid (ultra pure, 65 %) and 2 mL of perchloric acid (ultra pure, 70 %) in a CEM Mars 5 microwave oven. The digests were then diluted with deionised water to a total volume of 50 mL and analysed together with the filtered water samples for Fe, Mn and Zn using FAAS and As, Cd, Co, Cr, Cu, Ni, Pb and V using ETAAS with a GF3000 Graphite Furnace (AVANTA PM Atomic Absorption Spectrophotometer from GBC Scientific Equipment). All elements were assayed against the Atomic Absorption Standard Solution from Sigma Chemical Co. and blanks containing the same matrix as the samples. Results of element concentrations in the plants were calculated on a dry weight basis. The concentrations of As, Co, Cu, Cr, Ni and V in water were below the detection limits (μg L^−1^) of 1.5, 0.1, 0.1, 0.05, 0.5 and 1.5, respectively. The accuracy of the methods applied for the determination of element concentrations in plant samples was verified against Certified Reference Materials: moss M2 and M3 (Finnish Forest Research Institute). A coefficient of variance (CV) was calculated for the measured metal concentrations in the reference materials (Table 1).

**Table 1:** Analysis of certified reference material

Element	Moss M2 (Finnish Forest Research Institute) standards				Moss M3 (Finnish Forest Research Institute) standards			
	Certified	Found	Recovery	CV	Certified	Found	Recovery	CV
	(mg kg^−1^)		(%)		(mg kg^−1^)		(%)	
As	0.98 ± 0.007	0.96 ± 0.024	98.97	2.5	0.105 ± 0.007	0.107 ± 0.003	101.90	3.0
Cd	0.454 ± 0.019	0.460 ± 0.013	101.32	2.8	0.106 ± 0.005	0.109 ± 0.004	102.83	3.7
Co	0.98 ± 0.06	0.96 ± 0.02	97.96	2.4	0.115 ± 0.006	0.112 ± 0.002	97.39	2.4
Cr	0.97 ± 0.17	0.99 ± 0.02	102.06	2.0	0.67 ± 0.19	0.65 ± 0.02	97.01	3.1
Cu	67.7 ± 2.5	68.2 ± 1.4	100.74	2.1	3.76 ± 0.23	3.80 ± 0.10	101.06	2.8
Fe	262 ± 35	258 ± 11	98.47	4.4	138 ± 12	135 ± 5	97.83	3.5
Mn	342 ± 17	339 ± 9	99.12	2.6	535 ± 30	538 ± 16	100.56	3.1
Ni	16.3 ± 0.9	16.7 ± 0.3	102.45	1.8	0.95 ± 0.08	0.98 ± 0.02	103.16	2.3
Pb	6.37 ± 0.43	6.31 ± 0.22	99.06	3.6	3.33 ± 0.25	3.29 ± 0.10	98.79	3.2
V	1.43 ± 0.17	1.46 ± 0.08	102.09	5.3	1.19 ± 0.15	1.17 ± 0.07	98.32	5.6
Zn	36.1 ± 1.2	36.3 ± 0.7	100.55	1.9	25.4 ± 1.1	25.8 ± 0.7	101.57	2.6

## Statistical analysis

Differences between particular sampling sites in terms of element concentrations in water and mosses were evaluated by one-way ANOVA. The normality of the analysed features was verified using Shapiro-Wilk’s *W* test, and the homogeneity of variances was verified using the Brown-Forsythe test (Brown and Forsythe 1974; Argaç 2004). To obtain normal distribution of the features, data have been transformed with Box-Cox according to Zar (1999). Pearson correlations were calculated between the concentrations of elements in water and in moss tissues.

The matrix of concentrations of 11 elements (As, Cd, Co, Cr, Cu, Fe, Mn, Ni, Pb, V and Zn) in the moss samples collected from 48 sites downstream from the outlet in both rivers was subjected to numerical classification to identify groups of samples with similar patterns of metals. The clustering algorithm was prepared with complete linkage. The City-block (Manhattan) distance method was used for a similarity measure. Student’s *t* test was used to compare the concentration of trace elements between *F. antipyretica* groups as distinguished by cluster analysis.

The matrix of concentrations of 11 elements (As, Cd, Co, Cr, Cu, Fe, Mn, Ni, Pb, V and Zn) in the moss samples collected from 48 sites downstream from the outlet in both rivers was subjected to ordination to reveal possible gradients of concentration levels using principal component and classification analysis (PCCA). Plots of PCCA ordination of the plant samples and projection of element concentrations on the factor plane reveal similarities between the samples and correlations between the original variables and the first two factors (Legendre and Legendre 1998). The basis for PCCA is the principal component analysis often used in ecology to reduce the amount of data and stabilise subsequent statistical analyses (Vaughan and Ormerod 2005).

The contamination factor (CF) (Mouvet et al. 1986) was calculated as the ratio of metal concentration in the moss tested to the background level in that species established for the study area (the source area of the Nysa Łużycka river as investigated by Vázquez et al. (2004)). All calculations were done with Statistica 10 software (Statsoft. 2011).

## Results

The ranges of As and metal concentrations in water and moss samples are presented in Tables 2 and 3. The mean concentrations of elements in water and mosses differed significantly (ANOVA, *P* < 0.05).

**Table 2:** Minimum, maximum, mean and standard deviation (SD) of the concentration (mg L^−1^) of elements in water from the Nysa Łużycka and the Kwisa Rivers upstream and downstream from the sewage output; *t* test probability level (*P*) for comparison of both rivers

	Minimum	Maximum	Mean	SD	Minimum	Maximum	Mean	SD	*P*
Nysa Łużycka downstream					Kwisa downstream				
Cd	0.03	0.39	0.17	0.15	0.003	0.13	0.02	0.01	< 0.001
Fe	41	87	57	14	34	170	81	31	< 0.05
Mn	7	14	10	1.9	7	28	13	6	< 0.05
Pb	0.09	0.99	0.26	0.12	0.08	0.31	0.17	0.06	> 0.05
Zn	0.5	2.6	1.4	0.5	0.4	5.9	2.2	2.3	> 0.05
Nysa Łużycka upstream					Kwisa upstream				
Cd	0.03	0.04	0.04	0.02	0.007	0.01	0.009	0.002	< 0.001
Fe	30	34	32	1.1	32	36	34	1.8	< 0.05
Mn	6.8	7.6	7.1	0.3	6.1	6.9	6.7	0.3	< 0.05
Pb	0.08	0.12	0.10	0.01	0.06	0.19	0.10	0.05	< 0.05
Zn	0.9	1.2	1.1	0.1	0.3	0.6	0.3	0.03	< 0.001

**Table 3:** Minimum, maximum, mean and standard deviation (SD) of the concentration (mg kg^−1^) of elements in *F. antipyretica* from the Nysa Łużycka and the Kwisa Rivers upstream and downstream from the sewage output; *t* test probability level (*P*) for comparison of both rivers

	Minimum	Maximum	Mean	SD	Minimum	Maximum	Mean	SD	*P*
Nysa Łużycka downstream					Kwisa downstream				
As	12	22	17	2.8	10	26	16	5.1	> 0.05
Cd	3.2	9.1	5.8	1.9	2.4	6.1	3.8	1.2	< 0.001
Co	125	280	188	56	79	132	98	20	< 0.001
Cr	19	106	54	26	35	80	49 1	2	> 0.05
Cu	23	93	33	12	19	31	24	4	< 0.01
Fe	12,081	16,478	14,967	990	11,093	26,809	19,222	5350	< 0.01
Mn	15,160	30,234	21,359	4790	5130	16,160	10,172	4809	< 0.001
Ni	10	203	147	44	58	146	93	23	< 0.001
Pb	9	31	20	8	9	32	15	6	< 0.05
V	8	15	11	2	9	15	12	2	> 0.05
Zn	436	862	661	134	300	628	430	89	< 0.001
Nysa Łużycka upstream					Kwisa upstream				
As	10	12	11	0.5	6.5	7.4	6.9	0.3	< 0.001
Cd	3.1	3.9	3.5	0.3	1.8	2.3	2.1	0.2	> 0.05
Co	101	137	121	12	53	94	75	18	< 0.001
Cr	20	23	21	1.1	21	26	23	1.9	< 0.05
Cu	22	33	24	4.6	15	17	16	0.6	< 0.01
Fe	12,505	15,342	14,276	1219	8786	10,435	9754	660	< 0.001
Mn	14,220	17,140	15,990	1067	6120	7940	6813	660	> 0.05
Ni	74	88	80	6	71	93	79	5.7	> 0.05
Pb	8	11	10	0.9	6.3	8.0	7.2	0.5	> 0.05
V	7.3	8.0	7.8	0.3	6.3	7.7	6.8	0.5	< 0.01
Zn	450	494	473	16	247	459	344	104	< 0.05

The pH of streamwater ranged between 6.6 and 7.5 for the Nysa Łużycka and between 6.8 and 7.6 for the Kwisa, with the lowest results observed in the vicinity of sewage outputs (sites 1–3 and 13–15 for the Nysa Łużycka and sites 1–3 for the Kwisa). The pH is an important factor which influences trace element bioavailability by affecting speciation and properties of biological surfaces (Lithner et al. 1995). The authors also report that at pH < 6 (i.e. lower than in the rivers tested), the bioaccumulation factor in *F. antipyretica* decreases for Cd, Co, Ni and Zn.

The investigated rivers, both upstream and downstream from the sewage outputs, exceed the permitted values of trace element content in unpolluted surface water as determined by Kabata-Pendias (2001) (Cd < 0.005, Fe <0.5, Mn < 0.1 and Pb < 0.05 mg L^−1^). Elevated levels of these contaminants were observed also in the water samples collected upstream from the sewage outputs in both rivers, which can be attributed to an anthropogenic influence in the investigated areas (Vázquez et al. 2004). The concentrations of the elements downstream from the sewage outputs were significantly higher (*t* test, *P* < 0.05) than in the upstream samples for Cd, Fe and Mn in the Nysa Łużycka and for Fe, Mn and Pb in the Kwisa. The downstream part of the Nysa Łużycka contained significantly higher Cd concentrations and lower Fe and Mn concentrations than the respective section of the Kwisa (Table 2).

A comparison of trace element concentrations in *F. antipyretica* collected from upstream to downstream sites (*t* test, *P* < 0.05) revealed that the respective concentrations of As, Cd, Cr, Ni, Pb, V and Zn (the Nysa Łużycka) as well as As, Co, Cu, Fe, Pb and V (the Kwisa) were considerably higher (*t* test, *P* < 0.05) in the mosses downstream from the sewage outputs (Table 3) and significantly exceeded the concentrations (in parentheses, mg kg^−1^) of Co (3.6), Cr (4.1), Cu (10), Fe (3763), Mn (849), Ni (8.8), Pb (7.4), V (0.6) and Zn (130) against the background values given by Vázquez et al. (2004) for *F. antipyretica* collected from the source area of the Nysa Łużycka.

The calculation of the bioconcentration factor (BCF) from the metal content in the moss and the concentration in water leads to the following conclusions in terms of the accumulation properties of the plant investigated (Siebert et al. 1996). The ranges of BCFs for *F. antipyretica* for Cd, Fe, Mn Pb and Zn from the Nysa Łużycka are the following: 12–294, 161–371, 1342–3152, 36–331 and 212–1170, respectively, and from the Kwisa: 20–1484, 164–764, 276–2364, 40–207 and 103–931, respectively. These BCFs were lower than the values established by Martins and Boaventura (2002) for Cu (31,400) and Zn (4531) in the same species. The reason may be that the metal uptake rate tends to decrease with increasing metal exposure in water, which implies a toxic effect in mosses and subsequent deterioration of their physiological status (Martins and Boaventura 2002; Díaz et al. 2012).

**Fig. 2:**
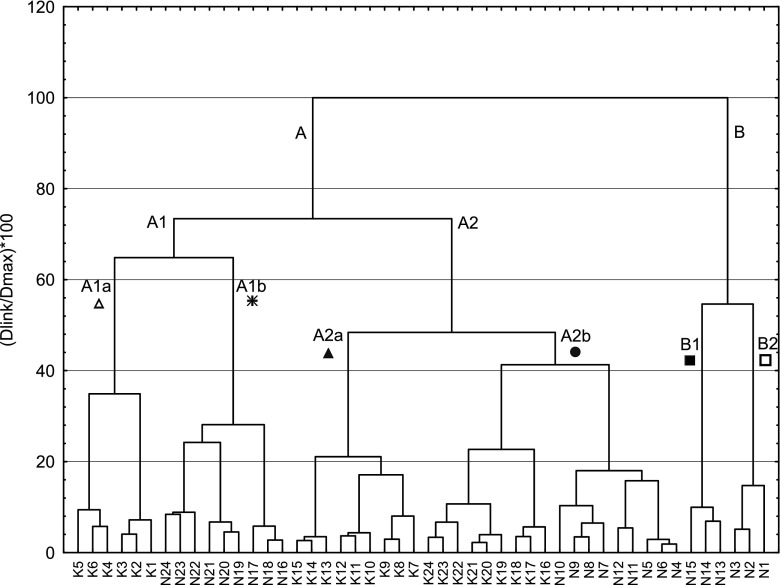
A diagram for the 48 cases based on 11 trace elements, with complete linkage clustering method; similarity method: city-block (Manhattan)

A dendrogram of the samples based on the concentration of the elements in *F. antipyretica* (Fig. 2) reveals similarities between particular sites. Two main clusters are formed: A (a grouping of Nysa Łużycka sites 4–12, 16–24 and all Kwisa sites) and B (a grouping of Nysa Łużycka sites 1–3 and 13–15). Cluster A is subdivided into two subclusters: A1 (Nysa Łużycka sites 16–24 and Kwisa sites 1–6) and A2 (Nysa Łużycka sites 4–12 and Kwisa sites 7–24); cluster B is also subdivided into two subclusters: B1 (Nysa Łużycka sites 13–15) and B2 (Nysa Łużycka sites 1–3). Subcluster A1 is further subdivided into two sub-subclusters: A1a (Kwisa sites 1–6) and A1b (Nysa Łużycka sites 16–24). Subcluster A2 is subdivided into two sub-subclusters: A2a (Kwisa sites 7–15) and A2b (Nysa Łużycka sites 4–12 and Kwisa sites 16–24). *F. antipyretica* collected from the sites in cluster A had a significantly lower concentration of As, Cd, Co, Cr, Cu, Mn, Ni and Zn (*t* test, *P* < 0.001) compared to plants from cluster B. This results from the fact that cluster B groups mosses collected from sites situated in the closest vicinity to the sewage outputs in the Nysa Łużycka. *F. antipyretica* included in cluster B1, affected by lignite industry sewage, had significantly higher concentrations of As, Cd, Co, Cr and Ni (*t* test, *P* < 0.001) than plants from cluster B2, which in turn contained significantly higher concentrations of Cu, Mn and Zn. Plants from cluster B2 grew in the mouth of the stream alimented by municipal sewage. Cu, Mn and Zn are typical components of roadside dust composed of gasoline exhausts, car components, oil lubricants and industrial and incinerator emissions (Wei and Yang 2010; Apeagyei et al. 2011; Nazzal et al. 2013). Additionally, fuel exhausts contain Mn which is often added as a combustion enhancer, smoke suppressant and to increase the octane rating (Lytle et al. 1994; Valavanidis et al. 2006).

**Fig. 3:**
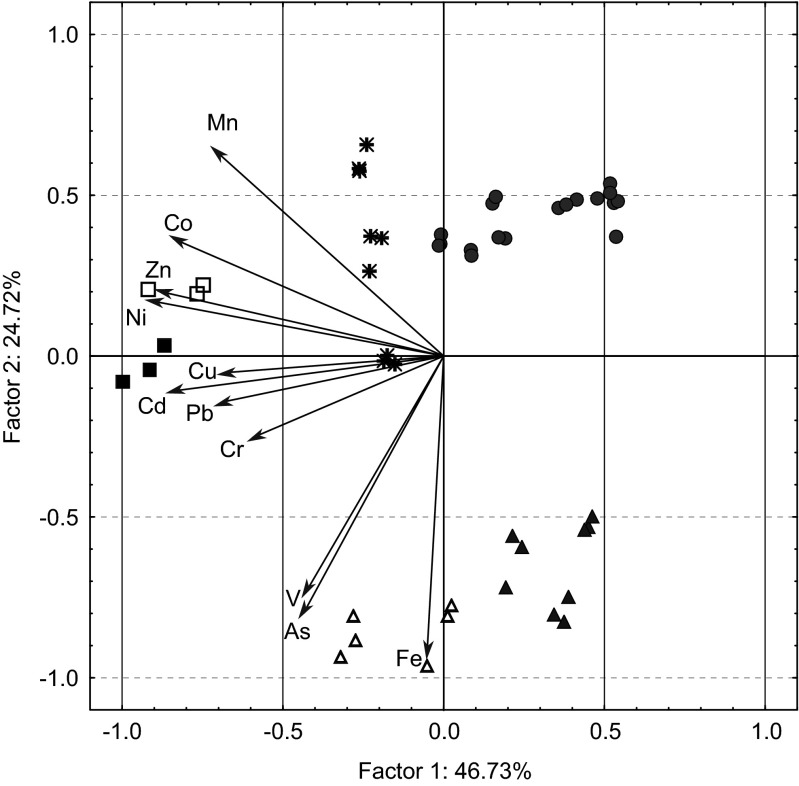
Ordination plot of *F. antipyretica* based on concentrations of the 11 elements: As, Cd, Co, Cr, Cu, Fe, Mn, Ni, Pb, V and Zn and projection of As and metal concentrations on the component plane. *Symbols* refer to the clusters, subclusters and sub-subclusters of Fig. 2

Figure 3 presents PCCA ordination indicating groups distinguished by the Joining tree. The first principal component discriminated between *F. antipyretica* from the Nysa Łużycka (sites 1–3, group B2 in the dendrogram; sites 13–15, group B1 in the dendrogram; and sites 16–24, group A1b in the dendrogram) and the Kwisa (sites 1–6, group A1a in the dendrogram) for which factor 1 returned negative scores, and sites 7–15 of the Kwisa (group A2a in the dendrogram), sites 4–12 of the Nysa Łużycka and 16–24 of the Kwisa (group A2b in the dendrogram) where factor 1 returned positive scores. Sites 1–3 were located in the closest vicinity of the sewage output, while sites 13–15 of the Nysa Łużycka were located in an area where the river was alimented by a stream polluted with municipal and industrial sewage. Additionally, *F. antipyretica* from the Kwisa (sites 1–6) situated nearest to the sewage output had negative scores of factor 2. The projection of the variables on the factor plane showed that *F. antipyretica* from sites 1–3 and 13–15 of the Nysa Łużycka had the highest tissue concentrations of Cd, Co, Cr, Cu, Mn, Ni, Pb and Zn. The moss from the Kwisa collected next to the sewage output had the highest concentrations of As, V and Fe, while *F. antipyretica* from sites 7–15 and 16–24 of the Kwisa and sites 4–12 of the Nysa Łużycka had the lowest concentrations of the elements. The latter sites were situated further downstream from the sewage output where the respective metal concentrations were already diminished in the course of the transport as a result of many processes such as dilution by ground and surface waters, mineral precipitation or sorption of elements onto precipitates and the streambed (Palmer et al. 2011).

## Discussion

Significant positive Pearson correlation was observed between the concentrations of Cd, Fe and Zn (*P* < 0.05) in the water and moss samples, while no such correlation was noted for Mn and Pb. An investigation by Say and Whitton (1983) reported the existence of significant positive correlations also between Mn and Zn concentrations in water and *F. antipyretica.* Siebert et al. (1996) calculated such relations between Cd and Zn concentrations in water and the species discussed here. However, Vázquez et al. (2004) observed no significant correlations between concentrations in water and in *F. antipyretica* with respect to most of the analysed elements. According to these authors, the bioavailability of metals to plants depends on a number of environmental factors and concentrations in water that usually show temporal variability. Mosses accumulate large quantities of metals in their cells during the period of high bioavailability in the environment. Once pollution has ceased and the environment is restored or cleaned up, the mosses start to release the previously accumulated metals, though at a slower rate compared to the accumulation rate during the phase of pollution (López et al. 1994; Vázquez et al. 2004). Metals concentrated in mosses are thought to reflect past metal levels in the environment (Yoshimura et al. 2000). Lithner et al. (1995) and Siebert et al. (1996) stated that correlations between certain metals in water and in *F. antipyretica* may lead to conclusive observations about the accumulation properties of this species.

According to Rasmussen and Andersen (1999), *F. antipyretica* is able to accumulate higher concentrations of Cu than of Cr. Copper is known to form complex bonds with organic materials and may thus more strongly bind with the moss than Cr (Rasmussen and Andersen 1999). In the present investigation, *F. antipyretica* was observed to accumulate more Cu than Cr only in the most polluted sites 1–3 of the Nysa Łużycka. These discrepancies may be caused by the fact that Rasmussen and Andersen’s investigation (1999) was carried out in brackish water. Martins et al. (2004) believe that *F. antipyretica* takes up much lower amounts of Zn than Cu. In the present study, the concentration of Cu was up to 32 times lower than that of Zn in the moss. Similar results, i.e. concentrations of Zn higher than of Cu in moss, were reported for *F. antipyretica* by Samecka-Cymerman et al. (2005). *F. antipyretica* in the examined streams yielded the highest BCF for Mn and Zn (up to 3152 and 931, respectively).

**Fig. 4:**
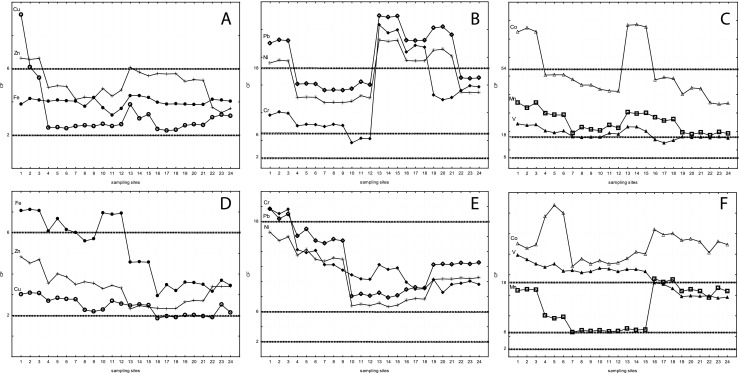
Contamination factors, CF (calculated as the ratio of metal concentration in *F. antipyretica* to the background level in that species) for metals in sites from the Nysa Łużycka (**a–c**) and the Kwisa (**d**, **e**); contamination classes by Mouvet et al. (1986): no contamination, CF < 2; suspected contamination, 2 < CF < 6; moderate contamination, 6 < CF < 18; severe contamination, 18 < CF < 54; extreme contamination, CF > 54

The trace element concentrations in *F. antipyretica* were used to determine the level of contamination in the examined rivers using the contamination factor (CF) (Mouvet et al. 1986; Vázquez et al. 2004). The CF for the upstream parts of both rivers showed clean sites (CF up to two times the background level or lower) for Pb, suspected pollution (2 < CF < 6) for Cu, Fe and Zn and moderate pollution (6 < CF < 18) for Co, Cr, Mn, Ni and V (Mouvet et al. 1986). The CF for downstream parts of both rivers revealed (Fig. 4) no clean sites. Extreme Co contamination (CF > 54) was detected in sites 1–3 and 13–15 of the Nysa Łużycka (Fig. 4), while all the other sites in this river can be classified with respect to this metal as severely polluted (18 < CF < 54) (Mouvet et al. 1986). For Mn, Ni, Pb and V, sites 1–3 in the Nysa Łużycka were severely contaminated (Fig. 4). The CF of Mn, Ni, Pb and V (sites 4–24, 13–21, 13–21, 4–6 and 12–15, respectively, in the same river) indicates severe pollution. Sites 1–3 in the Kwisa show severe contamination with Cr and Pb. Sites 1–24 can be classified as severely polluted with Co, while sites 1–15 as severely polluted with V. The calculated CF values indicated that both rivers were severely polluted with the metals mentioned above. The elevated element concentrations, as found in *F. antipyretica*, especially with respect to sites 1–3 in both rivers, i.e. those situated in the nearest vicinity of the sewage outputs, as well as site 13 and further downstream of the Nysa Łużycka, i.e. those situated at the mouth of the Witka stream, reflected the element composition of sewage produced by lignite, urban and glass sand industry. Our results are supported by the PCCA ordination (Fig. 3), which distinguished the *F. antipyretica* from the sites polluted with lignite industry sewage from that collected at the sites polluted with glass sand industry sewage. Elevated Cd, Co, Cr, Cu, Mn, Ni, Pb and Zn levels in samples of this species influenced by lignite industry are confirmed by Sarris et al. (2009), Suchara et al. (2011) and Jasion et al. (2013), all of whom reported these metals to be significantly accumulated in coal fly ashes emitted during lignite combustion. Elevated As, V and Fe levels in *F. antipyretica* influenced by glass sand industry sewage are in accordance with reports of Łuszczkiewicz (1987) and Muszer and Łuszczkiewicz (2006) on the Osiecznica deposit enriched with heavy minerals, including ilmenite, arsenopyrite, magnetite, hematite and pyrite. The Osiecznica deposits contain abundant ore minerals (simple sulphides which may be a source of undesired elements in products and intermediates).

This investigation contributes to a better understanding of the relation between the concentrations of elements in *F. antipyretica* and in water in an effort to evaluate the potential pollution in a river. The use of this particular species is very important in bioindication as the pattern of variability of elements in *F. antipyretica* can be used to confirm the type of pollution.

## Conclusion

1. The Nysa Łużycka and the Kwisa rivers were heavily polluted with As, Cd, Co, Cr, Cu, Fe, Mn, Ni, Pb, V and Zn, which were reflected in the extremely high concentration of these elements in the locally growing *F. antipyretica*.
2. *F. antipyretica* from the river receiving lignite industry sewage accumulated the highest concentrations of Cd, Co, Cr, Cu, Mn, Ni, Pb and Zn in its tissues, while that from the river influenced by glass sand industry sewage accumulated the highest concentrations of As, V and Fe.
3. The principal component and classification analysis classifies the concentration of elements in the aquatic moss *F. antipyretica*, which enables differentiation between sources of pollution in the rivers in particular areas of mining industry.
4. The results of this investigation permit conclusions as to the water pollution level in both rivers on the basis of the concentrations of trace elements observed in *F. antipyretica.*
